# Clinical Characteristics and Outcomes of Patients with Positive Celiac Serology and Cancer Therapy Exposure

**DOI:** 10.7150/jca.63837

**Published:** 2022-01-01

**Authors:** Barbara Erthal Smeral Dutra, Dongguang Wei, Dongfeng Tan, Mazen Alasadi, Hao Chi Zhang, Austin Thomas, Anusha Shirwaikar Thomas, David Matthew Richards, Yinghong Wang

**Affiliations:** 1 Department of Internal Medicine, The University of Texas Health Science Center, 6431 Fannin Street, MSB 1.134, Houston, TX 77030, USA.; 2 Department of Pathology, The University of Texas MD Anderson Cancer Center, 1515 Holcombe Blvd., Unit 1466, Houston, TX 77030, USA.; 3 Department of Pathology and Laboratory Medicine, University of California Davis Medical Center, 4400 V Street, Sacramento, CA 95817, USA.; 4 Department of Gastroenterology, Hepatology & Nutrition, The University of Texas MD Anderson Cancer Center, 1515 Holcombe Blvd., Unit 1466, Houston, TX 77030, USA.

**Keywords:** celiac disease, chemotherapy, diarrhea, immunomodulator therapy, malnutrition

## Abstract

**Objectives:** The prevalence of celiac disease (CD) among cancer patients is unknown, yet new cases of CD occur after cancer therapy exposure. The aim of this study was to describe the clinical course and endoscopic features of patients with positive celiac serology (PCS) post-cancer therapy exposure (PCTE) as compared to those with no cancer therapy exposure (NCTE).

**Methods:** A retrospective study of adult patients with PCS at MD Anderson Cancer Center between March 2009 and May 2020. Patients with positive tTG IgA, tTG IgG, and/or EMA IgA were categorized into cases with NCTE and PCTE. Clinical course, endoscopic and histologic features, and treatments were compared between the two groups.

**Results:** Of the 4,345 patients screened for celiac serology, 21 (0.5%) met inclusion criteria. 12 were PCTE, with a median time of 258 days (173-930 days) from initiation of the last cancer therapy. Those PCTE had a higher rate of diarrhea (75% vs 22%, p = 0.030), malnutrition and death. A gluten-free diet was initiated in 82% PCTE vs 89% NCTE, with the majority experiencing symptom resolution. There were no significant differences in endoscopic and histologic features. 17 patients met criteria for CD diagnosis.

**Conclusions:** Our findings suggest that CD may be under-diagnosed in cancer patients. Patients with PCS after cancer therapy may present with diarrhea, nutritional deficiencies, and malnutrition, yet a gluten-free diet may be efficacious in treatment management. Therefore, CD should be considered when treating cancer patients. Given the relative proximity of PCS to cancer therapy exposure, future studies should investigate the association of cancer and cancer therapy with the development of CD.

## Introduction

Celiac disease (CD) is a systemic immune-mediated disease that leads to small bowel malabsorption after exposure to dietary gluten in genetically predisposed people 1. Although gluten is a known environmental trigger for CD, the time from gluten exposure to the development of autoantibodies and clinical manifestations of CD is variable. It is unclear what other environmental factors can trigger the development of CD, current studies have reported a possible association with a history of gastrointestinal infections, gluten exposure in early age, medications and more recently changes in the gut microbiome 2-4. At this time, data is still lacking regarding the association between cancer, cancer therapy and the development of CD, especially in a genetically susceptible population. Patients with CD commonly present with fatigue, weight loss, diarrhea, and nutritional deficiencies due to malabsorption as a result of autoimmune destruction of the intestinal villi in the presence of gluten 5,6. CD diagnosis is based on serology and biopsy of the duodenum with the ultimate treatment being a gluten free diet 7.

Diarrhea is also a common adverse effect of cancer therapy, with severe grade 3 to 4 diarrhea occurring in up to 47% of patients undergoing chemotherapy, this can affect cancer treatment and ultimately survival 8,9. The pathophysiology of chemotherapy-induced diarrhea is not fully understood but thought to be multifactorial, from mucosal injury resulting in ulceration and atrophy to alterations in enzymatic function and changes in the microbiome 9,10. Chemotherapy also leads to immunosuppressive and immunomodulatory effects 11, which may be implicated in the immunologic and inflammatory damage that occurs in the small intestine in the presence of gluten 12. Consequently, although unproven, it remains plausible that cancer therapy may promote gut mucosal autoimmunity.

CD diagnosed after exposure to cancer therapy has been reported in the literature after evaluation of refractory diarrhea, with diarrhea improving and resolving after initiation of a gluten-free diet based on limited evidence 13-15. Furthermore, the true prevalence and natural history of CD in patients being treated for cancer is unknown, as is the effect of CD on cancer treatment and long-term outcomes.

We performed a retrospective descriptive study on the clinical course, endoscopic features, and outcomes of cancer patients with positive celiac serology (PCS) with and without cancer therapy exposure.

## Materials and methods

### Study Design and Population

This retrospective, descriptive, single-center study included adult patients with PCS at The University of Texas MD Anderson Cancer Center between March 2009 and May 2020. The study was conducted with approval from the Institutional Review Board at MD Anderson. Informed consent from patients was waived for this study. We identified patients aged 18 years or older who tested positive for anti-tissue transglutaminase (tTG) immunoglobulin A (IgA) antibody with a value greater than 10 U/mL, tTG immunoglobulin G (IgG) antibody with a value greater than 9 U/mL, and/or positive anti-endomysial (EMA) IgA antibody. These test were conducted with Inova Diagnostics' ELISA testing. Patients were excluded if they had received a transplant or had been diagnosed with gastrointestinal graft-versus-host disease.

Patients were categorized into two groups: patients with PCS with no cancer therapy exposure (NCTE) and those with PCS post-cancer therapy exposure (PCTE). In addition to the tTG IgA, tTG IgG and EMA IgA celiac serologies, we collected data on deaminated gliadin peptide (DGP) IgG and IgA antibodies (ELISA test by Inova Diagnostics) and the genetic haplotypes of HLA-DQ2 and HLA-DQ8 when available. CD diagnosis was defined as PCS with histologic findings from esophagogastroduodenoscopy (EGD) duodenal biopsies consistent with CD including intraepithelial lymphocytosis, crypt hyperplasia, and villous atrophy 1. The protocol at MD Anderson for the endoscopic assessment for CD is in alignment with the standard protocol for celiac biopsy sampling.

Patients demographic information, medical, and oncologic history were collected. Medical history included comorbid conditions, history of autoimmune diseases, family history of CD and use of proton pump inhibitors (PPIs), nonsteroidal anti-inflammatory drugs (NSAIDs), or steroids. Oncologic history included cancer type and staging. Cancer therapy was classified as chemotherapy, hormonal or immunomodulator therapy. Cancer therapy-related adverse events, mortality, and cause of death were also collected.

CD related manifestations, endoscopic evaluation, treatments and outcomes were collected. Clinical manifestations included diarrhea, weight loss, and nutritional deficiencies. Severity of diarrhea was defined based on the National Cancer Institute's Common Terminology Criteria for Adverse Events version 5 (CTCAE) 16. EGD features suggestive of CD included the presence of mucosal changes such as scalloping, fissures, nodularity, atrophy, ulceration and non-ulcerative inflammation including erythema, friability, erosions, inflammatory exudate, loss of vascular pattern, and edema. Histologic features included intraepithelial lymphocyte infiltration, crypt hyperplasia, villous blunting and atrophy. Treatment including a gluten-free diet, antidiarrheal medications, and immunosuppressant agents were reported. Outcomes included symptom resolution, hospitalization, need for nutritional support and mortality.

### Statistical analysis

A descriptive statistical analysis was performed. The distribution of continuous variables was summarized by using means and standard deviations. The distribution of categorical variables was summarized by using frequencies and percentages, and the associations of these variables were evaluated with the Fisher exact test. All statistical evaluations were 2-sided, and p values of less than 0.05 were considered statistically significant.

## Results

**Figure 1:**
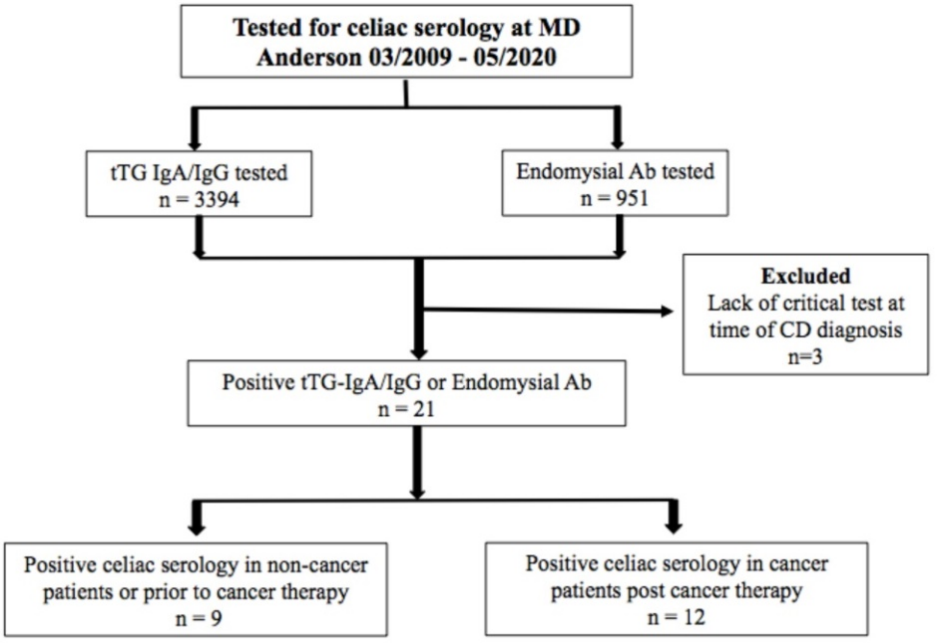
Patient allocation flowchart.

Between March 2009 and May 2020, 4,345 patients at MD Anderson were tested for tTG IgA antibody, tTG IgG antibody, and/or EMA IgA antibody. 21 patients (0.5%) met the inclusion criteria for PCS (Figure 1). Of the 21, 15 (71%) were cancer patients; 12 (57%) were PCTE and 9 (43%) had NCTE. Of the patients screened, 17 (0.4%) met criteria for CD diagnosis; 9 were PCTE and 8 had NCTE.

### Patient Characteristics

Patients with PCS in the PCTE group were significantly older than those with NCTE (58 vs 47 years, p = 0.019) (Table 1). Although not significant, the PCTE group were predominantly male (58%) and had higher rates of comorbidities (83%), but similar rates of autoimmune diseases (17%) and family history of CD (17%). Only 1 case had a co-infection at the time of endoscopic evaluation, which was Helicobacter *pylori* in the NCTE group.

### Oncologic History

Of the 9 NCTE patients, 3 were diagnosed with stage I or II solid tumors prior to PCS (Table 1). Of the 12 PCTE patients, 11 (92%) had solid tumors and 83% of the cases had stage III or IV cancer at the time of PCS. The 12 PCTE patients had PCS at a median of 875 days (336-2572 days) after cancer diagnosis, 258 days (173-930 days) after initiation of the last cancer therapy, and 117 days (1-393 days) after the last cancer therapy exposure (9 had PCS within 1 year of the last cancer therapy exposure). All 12 patients were exposed to more than one cancer therapy agent. 11 of the 12 cases were exposed to chemotherapy, 5 cases were exposed to immunomodulator therapy and 1 case had hormone therapy exposure (Table 2).

### Celiac Serology

All 9 patients with NCTE had positive tTG IgA, with 3 having an additional PCS. 11 of 12 PCTE patients tested positive for tTG-IgA with 5 having more than one PCS test. Additional celiac serology included tTG IgG antibody, EMA IgA antibody and DPG IgA or IgG antibody. Only 4 patients, all in the PCTE group, had HLA DQ2 and DQ8 available, with 3 of the 4 being positive.

### Celiac-related Symptoms

Diarrhea was more frequently reported among PCTE patients than in NCTE (75% vs 22%, p = 0.030). Although not statistically significant, PCTE patients had a lower rate of reported nutritional deficiencies (67% vs 78%) but higher rates of weight loss (50% vs 11%) and malnutrition (25% vs 0%) (Table 3).

### Endoscopic and Histologic Features

**Figure 2:**
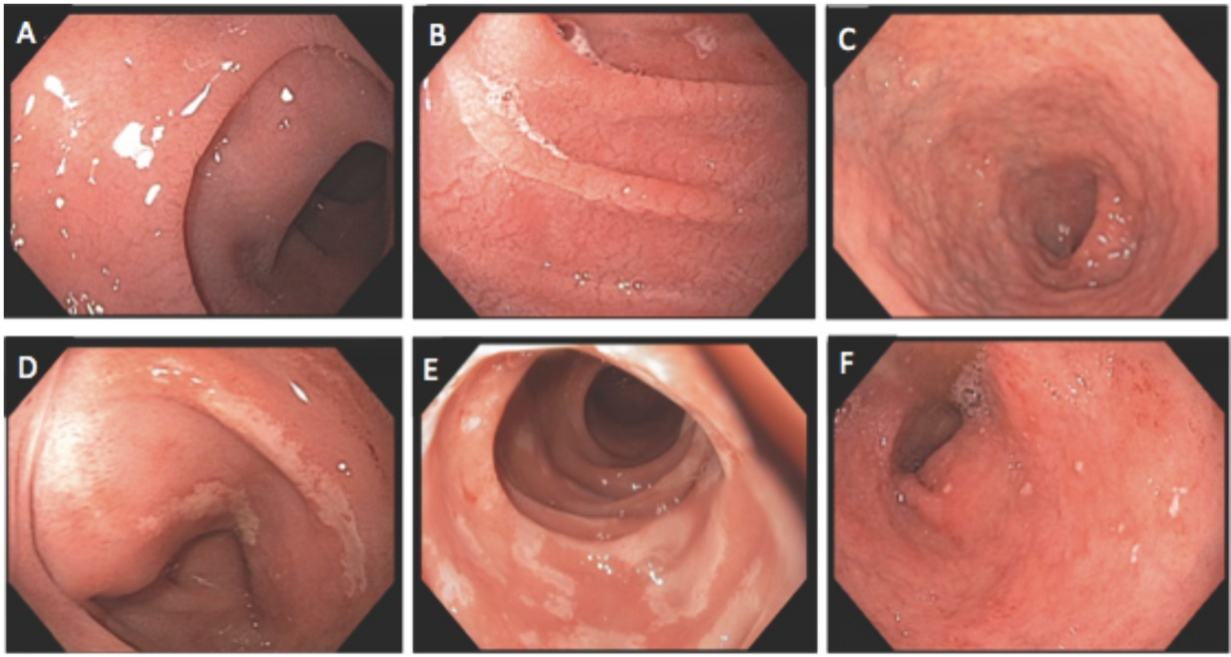
Endoscopic findings of duodenum: **A and B)** Mucosal scalloping; **C)** mucosal atrophy; **D and E)** mucosal erosion; **F)** mucosal erythema with small aphtha.

**Figure 3:**
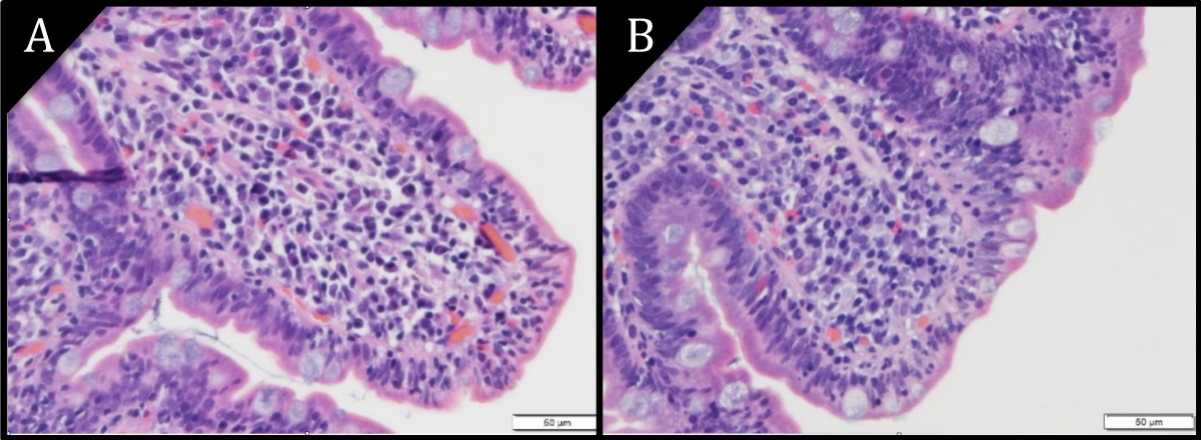
Pathology images of duodenal epithelium (H&E) at magnification 40X. **A)** Villous atrophy. **B)** Villous blunting. Increased epithelial lymphocytes are seen in both images.

Of the 21 patients with PCS, 20 had EGD results available, with all but 1 EGD performed within 6 months of PCS. Eight of nine NCTE patients had an EGD with duodenal biopsies, of these, 7 had abnormal endoscopy features, while all had abnormal histology (Table 2). One patient was initiated on a gluten-free diet 5 months before the EGD. All 12 PCTE patients underwent EGD, and 10 had duodenal biopsies. Of these 12, 7 had normal duodenal mucosa, yet all 10 with biopsy had abnormal histology. One patient was initiated on a gluten-free diet 3 months before the EGD, and 8 were exposed to cancer therapy within 12 months of the EGD. Of the 12 PCTE patients, those with normal and abnormal EGD findings had similar exposure to the type of cancer therapy, occurrence of diarrhea, and resolution of diarrhea on a gluten-free diet (Table 4). Endoscopic and histologic images are shown in Figures 2 and 3.

### Treatment and Outcome

Nearly all of the patients with diarrhea at the time of evaluation had symptom resolution on a gluten-free diet. Antidiarrheal medication was administered to seven patients and systemic steroid to one patient in the PCTE group. Of the six patients who received cancer therapy at the time of PCS, three had therapy held due to diarrhea. Other cancer therapy-related GI adverse effects such as pancreatitis and liver toxicity were documented in 6 of 12 patients with PCTE, yet none were diagnosed with ileitis or colitis. Only one patient (in the PCTE group) had severe malnutrition necessitating hospitalization and treatment with total parenteral nutrition and systemic steroid.

A total of 4 patients, all in the PCTE group, died during the study window. All deaths were related to underlying malignancy, with one case associated with superimposed fungemia and multiorgan failure related to prolonged total parenteral nutrition therapy for severe malnutrition.

## Discussion

To date, there are limited data on the clinical course of Celiac Disease in cancer patients. Although cancer therapy is known to cause systemic changes, it remains unclear whether it triggers the autoimmunity seen in CD. The findings from this study suggest that screening for CD remains important when evaluating patients with cancer therapy exposure who present with gastrointestinal symptoms and nutritional deficiencies given the unique and efficacious treatment of CD with a gluten-free diet.

Currently, the prevalence of CD in cancer patients is unknown. In this study of 4,345 patients with manifestations suggestive of CD who completed serologic screening, approximately 0.5% had positive serology and 0.4% met criteria for CD diagnosis. This is lower than the estimated prevalence of CD in the general population, which is 0.7%-1.0% in the United States 17,18. We suspect the lower rate of CD in our cohort to be multifactorial. The primary manifestations of CD that prompted celiac screening among PCTE patients were nutritional deficiencies, weight loss, and diarrhea, however these do not encompass all the manifestations of CD. Furthermore, recent studies have reported that the collective symptoms of CD aside from diarrhea outnumber the cases presenting with diarrhea 4. Diarrhea, nutritional deficiencies and weight loss also overlap with various conditions including cancer itself, adverse effects of cancer therapy and other co-morbidities. It is therefore possible that not all patients with these manifestations on cancer therapy have been screened for CD at MD Anderson. Of clinical importance, the findings of nutritional deficiencies, weight loss, and malnutrition in a mostly older patient population correlated with the rising recognition of these findings as CD manifestations among the elderly 19-21. Yet, studies continue to demonstrate that CD is often missed in the elderly population and therefore there should be a high index of suspicion for CD in these patients 22. Lastly, not all patients in our study had biopsy for histologic review. While our study raises concerns for the need to consider CD in cancer patients, given the retrospective and single center design of this study, our cohort does not provide a full representation of the manifestations of CD or the prevalence of CD amongst patients with cancer. For the aforementioned reasons, we suspect our cohort study likely underestimates the prevalence of CD at our institution. Future prospective studies are warranted to further clarify the prevalence, clinical management of CD and its outcome among cancer patients on therapy if clinical manifestations raise the suspicion of CD.

While the association between CD and the development of cancer has been investigated, the prevalence of malignancy before and after CD diagnosis is unknown. In one meta-analysis, those with CD were at increased risk of developing cancer with an odds ratio of 1.25 23. Rampertab et al. 5 reported malignancy in 9.3% of CD patients, with 64% diagnosed with cancer before and 27% after CD diagnosis. In our study, 71% of patients had a cancer diagnosis before PCS, however our study has inherent selection bias being at a cancer center. In a recent study of 47,241 patients with CD, there was a 1.11 fold increased risk of cancer which was primarily encountered within the first year after CD diagnosis and primarily involved hematologic and gastrointestinal cancers 24. This increased risk of cancer including gastrointestinal cancers are also supported by other studies 23,25. We found that 50% of the PCTE group in our cohort had gastrointestinal malignancies including colon, esophageal and hepatic cancers. However, similar to Lebwohl et al., we are unable to determine whether these patients had gastrointestinal malignancies in the setting of unrecognized CD or developed CD after cancer diagnosis or cancer therapy exposure.

Small bowel histologic features such as intraepithelial lymphocytosis and villous atrophy are hallmark findings for CD 26,27. Yet, these findings are present in other conditions including infections, autoimmune diseases, radiation injuries, and drug-induced injuries such as those from NSAIDS, PPIs, and chemotherapy 28-33. While 14 of 21 cases had other risk factors for gastrointestinal injury (cancer therapy, PPI, NSAIDs, H*. pylori*), they all had PCS and most experienced clinical improvement on a gluten-free diet. Furthermore, the presence of histologic abnormalities was not associated with endoscopic duodenal findings. Endoscopic features have a sensitivity of 82%-94% for CD-related histologic abnormalities in patients suspected of having CD compared to 59%-88% in those undergoing EGD for any reason 34-37. In our study, 87% of NCTE patients and 42% of PCTE patients had abnormal endoscopic features despite all patients having abnormal histologic findings. This illustrates the importance of obtaining endoscopic biopsies.

Of particular clinical relevance is the association between cancer therapy and CD manifestations. To date, there are limited data on CD in patients post cancer therapy exposure. Of our 21 cases, 12 (57%) were PCTE. They were older, had more advanced cancers, and exposure to various cancer therapies, although no specific cancer therapy was found to be associated with PCS. Furthermore, PCS occurred at a median time of 875 days after cancer diagnosis and 258 days after initiation of last cancer therapy. Interestingly, Robinson et al. 13 found that 12 out of 27 cancer patients were diagnosed with CD after cancer therapy. Recent data have also demonstrated an association between CD and exposure to immune checkpoint inhibitors, which are used in various cancer treatments 15, 38-42. Immune checkpoint inhibitors cause an upregulation of the immune system with studies showing inflammatory changes in duodenum that are distinct from celiac disease. Interestingly there are reports of new diagnosis of CD post immune checkpoint inhibitor exposure requiring a gluten free diet for treatment as well as duodenitis with negative celiac serology testing however with response to gluten free diet after failed steroid and biologic therapy. While in our study there was only one case of CD post immune checkpoint inhibitor treatment, larger studies are needed to evaluate if there is a causal relationship. In addition to the potential immunologic changes in the gut mucosa associated with cancer therapy, studies have also raised the concern that cancer therapy can alter the microbiome and there is evidence suggesting the potential role of the microbiome in the pathogenicity of CD 10, 24. Given the relative proximity of PCS to cancer therapy and the similarity of histologic changes from cancer therapy to CD, future research are needed to determine whether cancer or cancer therapy can incite environmental changes that can lead to CD in predisposed patients, a celiac-related enteropathy with PCS, or if therapy is simply unmasking undiagnosed CD.

While CD manifestations overlap with the adverse effects of cancer therapy, most patients had resolution of diarrhea and nutritional deficiencies after initiation of a gluten-free diet. This raises the need to consider CD and possible gluten-sensitivity during cancer therapy. Also of clinical significance is that malnutrition in cancer patients may range from 30% to 90% and is associated with inferior response to therapy and survival 43,44. There was one death in the PCTE group associated with malnutrition, highlighting the importance of considering CD-associated malabsorption in exacerbating the adverse effects of cancer treatment and ultimately mortality. Lastly, it is imperative to further evaluate the role of CD in cancer patients as there are newer medical therapies being studied such as transglutaminase 2 inhibitors which may be of value in cancer patients who are already at risk for malnutrition and decreased food intake 45.

Our study had certain limitations. Its retrospective nature, single-center study design, and small sample size lacked the power to ascertain the prevalence and incidence of CD among cancer patients at our institution and to make definitive conclusions. Given multiple confounding factors related to cancer and cancer therapy and cases lacking histology, definitive diagnoses of CD could not be made for all cases. Furthermore, the overlapping manifestations of CD and the adverse effects of cancer therapy and advanced malignancy, it is likely that not all patients at risk for CD or with CD manifestations were actually screened for CD. Given the small cohort number and inability to determine the true prevalence of CD, we were unable to make definitive statements on the sensitivity, specificity and predictive values of serology testing on this patient population. Finally, the control group is a biased patient population at a cancer institution and do not represent the general population with CD. Despite our limitations and inability to determine causality, our findings expand on the limited data available regarding the potential effect that cancer therapy may have on CD.

## Conclusions

Our findings suggest that CD may be underdiagnosed in cancer patients and that patients with PCS after cancer therapy may present with diarrhea, nutritional deficiencies, and malnutrition. Given the unique and efficacious treatment of CD with a gluten-free diet, clinicians should consider CD when evaluating cancer patients with gastrointestinal symptoms and nutritional deficiencies. Future studies are warranted to determine the prevalence of CD in cancer patients and the association of cancer therapy with the development of CD.

## Figures and Tables

**Table 1:** Demographics and Cancer Characteristics of Patients with Positive Celiac Serologies Stratified by Cancer Therapy Exposure

Characteristics at time of CD	No. Patients (%)		*p*
	NCTE, n = 9 (%)	PCTE, n = 12 (%)	
Age^ a^, mean (SD), y	47 (10.5)	58 (8.5)	0.019
Male sex	4 (44)	7 (58)	0.523
Female sex	5 (55)	5 (42)	-
Caucasian race	7 (78)	10 (83)	0.670
Black	0 (0)	1 (08)	1.000
Hispanic	2 (22)	0 (0)	0.172
Asian	0 (0)	1 (08)	1.000
Co-morbidities	5 (56)	10 (83)	0.331
Other autoimmune diseases	2 (22)	2 (17)	<0.001
Family history of celiac disease	0 (0)	2 (17)	0.486
**Risk Factors**			
NSAID^ b^	1 (11)	1 (8)	1.000
PPI ^b^	1 (11)	5 (42)	0.178
Steroid ^b^	0 (0)	1 (08)	1.000
H. *pylori* infection at the time of celiac diagnosis	1 (11)	0 (0)	0.429
**Cancer ^c^**			
Solid	3	11 (92)	-
Head and neck	1	0 (0)	-
Breast	0	5 (42)	
GI	-	6 (50)	-
Melanoma	1	0 (0)	-
Skin	1	0 (0)	-
Hematologic	-	1 (8)	-
No cancer	6 (67)	0 (0)	-
**Cancer Stage**			
≤ II	3 (33)	2 (17)	-
III/IV	0 (0)	10 (83)	-

**^a^** Mean age at Diagnosis of Positive Celiac Serology; ^b^ Therapy used within 3 months prior to EGD at time of celiac screening or positive serology if no EGD available; ^c^ For the NCTE group, 3 cases were diagnosis with solid cancers including head and neck, skin and melanoma however none of these cases received cancer therapy prior to CD diagnosis; Abbreviations: NCTE: No cancer therapy exposure; PCTE: post cancer therapy exposure; GI: gastrointestinal; GU: genitourinary.

**Table 2:** Description of the Last Cancer Therapy Received prior to Positive Celiac Serology in Patients Post Cancer Therapy Exposure

Therapy	No. Patients (%), PCTE, n = 12
**Chemotherapy**	11 (92)
Antimicrotubule Agents (docetaxel, paclitaxel, vincristine)	5 (42)
Topoisomerase inhibitors (Irinotecan)	1 (8)
Anti-metabolites (5-fluorouracil, capecitabine)	5 (42)
Anthracyclines (doxorubicin, epirubicin)	4 (33)
Alkylating Agents (carboplatin, cisplatin, oxaliplating, cyclophosphamide)	9 (75)
Kinase Inhibitors (lenvatinib**^a^**, dabrafenib**^b^**)	2 (17)
**Immunomodulator therapy**	5 (12)
Lenolidomine, rituximab, bevacizumab, trastuzumab, pentuzumab, panitumumab, nivolumab	
**Hormone Therapy**	1 (8)
Leuprolide, tamoxifen	

**^a^** VEGF tyrosine kinase inhibitor; **^b^** BRAF kindase inhibitor; A total of 10 patients out of 12 were on multiple agents; Abbreviations: PCTE: post cancer therapy exposure.

**Table 3:** Clinical Characteristics at the Time of Positive Celiac Serologies Stratified by Cancer Therapy Exposure

Characteristics	NCTE, n = 9	PCTE, n = 12	*P*
**Symptoms**	n = 9 (%)	n = 12 (%)	
***Diarrhea***	2 (22)	9 (75)	0.030
Grade 1-2 (≤ 6 BM/day above baseline)	1	7	
Grade 3-4 (> 6 BM/day above baseline)	1	2	
Nutritional deficiencies ^a^	7 (78)	8 (67)	0.659
Weight loss	1 (11)	6 (50)	0.087
Malnutrition diagnosis	0 (0)	3 (25)	0.229
**Celiac Diagnosis**	8 (89)	9 (75)	0.603
***EGD Features***	n = 8 (%)	n = 12 (%)	
Normal	1 (13) ^b^	7 (58)	0.067
Inflammatory changes	3 (33) ^b^	3 (25)	1.000
Scalloping, fissures, nodularity	3 (38) ^b^	3 (25)	0.642
Atrophy	2 (25) ^b^	1 (8)	0.537
**Histology Features**	n = 8 (%)	n = 10 (%)	
Intraepithelial lymphocytosis	8 (100) ^b^	9 (90) ^c^	1.000
Crypt hyperplasia	1 (13) ^b^	1 (10) ^c^	1.000
Villous atrophy	8 (100) ^b^	8 (80) ^c^	0.477
**Treatment**	n = 9 (%)	n = 12 (%)	
Gluten-free diet	8 (89)	9 (82)	0.603
Anti-diarrheal	0 (0)	7 (64)	0.007
Steroids	0 (0)	1 (08)	1.000
**Symptom Outcomes ^d^**			
Diarrhea resolved	n = 2 (%), 2 (100)	n = 9 (%), 8 (89)	1.000
Nutritional deficiency resolved	n = 7 (%), 6 (86)	n = 8 (%), 5 (63)	1.000
Hospitalization	n = 9 (%), 0 (0)	n = 12 (%), 1 (08)	1.000
Median follow-up duration (IQR), y	7.1 (6.0-8.5)	3.8 (0.9-5.3)	<0.001
Overall mortality	n = 9 (%), 0 (0)	n = 12 (%), 4 (33)	0.104

^a^ Nutritional deficiencies included micronutrient deficiencies, such as iron deficiency and vitamins deficiencies; ^b^ EGD and histological features are of the 8 cases in which EGD and histology reports were available among the 9 patients with NCTE; ^c^ Histological features are of the 10 reports available from the 12 PCTE patients who underwent EGD; ^d^ The percent resolution is the proportion of the patients with symptoms; Multiple symptoms can be presented among same patients. Abbreviations: EGD, esophagogastroduodenoscopy; IQR, Interquartile Range.

**Table 4:** Characteristics of Positive Celiac Serology Cases in the Post Cancer Therapy Exposure Group Stratified by EGD Findings

Characteristics	Normal EGD^a^, n = 7	Abnormal EGD^b^, n = 5
**Symptoms**	n = 7 (%)	n = 5 (%)
Diarrhea	5 (71)	5 (100)
Nutritional deficiencies	3 (43)	4 (80)
Weight loss	4 (57)	2 (40)
**Histology Features^ c^**	n = 5 (%)	n = 5 (%)
Intraepithelial lymphocytosis	5 (100)	4 (80)
Crypt hyperplasia	0 (0)	1 (20)
Villous atrophy	4 (80)	4 (80)
**Cancer Therapy Exposure**	n = 7 (%)	n = 5 (%)
Chemotherapy	6 (86)	5 (100)
Hormone therapy	1 (14)	0 (0)
Immunomodulator therapy	3 (43)	2 (40)
Any cancer therapy **^d^**	5 (71)	3 (60)
**Other cancer therapy related GI adverse effects ^e^**	5 (71)	1 (20)
**Symptom Outcomes^ f^**		
Diarrhea resolved	n = 5 (%), 5 (100)	n = 5 (%), 4 (80)
Nutritional deficiency resolved	n = 3 (%), 2 (67)	n = 4 (%), 2 (50)
Hospitalization	n = 7 (%), 0 (0)	n = 5 (%), 1 (20)

**^a^** Normal EGD refers to endoscopic duodenal evaluation; **^b^** Abnormal EGD refers to duodenal evaluation with findings reported as inflammation, scalloping, fissures, nodularity or atrophy; **^c^** Percentages are out of 5 cases that had duodenal biopsy with normal EGD features and 5 cases that had duodenal biopsy with abnormal EGD features; **^d^** Patients who received any cancer therapy within 12 months of positive celiac serology; **^e^** Other cancer therapy related GI adverse effects included liver toxicity, pancreatic toxicity, proctitis and oral mucositis. No cases were diagnosed with ileitis or colitis; ^f^ The percent resolution is out of the patients with symptoms; Multiple symptoms and histology features can be presented among same patients.

## References

[1] Green PH, Cellier C (2007). Celiac disease. N Engl J Med.

[2] Fasano A, Catassi C (2012). Clinical practice. Celiac disease. N Engl J Med.

[3] Catassi C, Kryszak D, Bhatti B (2010). Natural history of celiac disease autoimmunity in a USA cohort followed since 1974. Ann Med.

[4] Lebwohl B, Rubio-Tapia A (2021). Epidemiology, presentation, and diagnosis of celiac disease. Gastroenterology.

[5] Rampertab SD, Pooran N, Brar P (2006). Trends in the presentation of celiac disease. Am J Med.

[6] Lo W, Sano K, Lebwohl B (2003). Changing presentation of adult celiac disease. Dig Dis Sci.

[7] Husby S, Murray JA, Katzka DA (2019). AGA Clinical Practice Update on Diagnosis and Monitoring of Celiac Disease-Changing Utility of Serology and Histologic Measures: Expert Review. Gastroenterology.

[8] Rothenberg ML, Meropol NJ, Poplin EA, Van Cutsem E, Wadler S (2001). Mortality associated with irinotecan plus bolus fluorouracil/leucovorin: summary findings of an independent panel. J Clin Oncol.

[9] Andreyev J, Ross P, Donnellan C (2014). Guidance on the management of diarrhoea during cancer chemotherapy. Lancet Oncol.

[10] Di Fiore F, Van Cutsem E (2009). Acute and long-term gastrointestinal consequences of chemotherapy. Best Pract Res ClinGastroenterol.

[11] Heinhuis KM, Ros W, Kok M (2019). Enhancing antitumor response by combining immune checkpoint inhibitors with chemotherapy in solid tumors. Ann Oncol.

[12] Maiuri L, Ciacci C, Ricciardelli I (2003). Association between innate response to gliadin and activation of pathogenic T cells in coeliac disease. Lancet.

[13] Robinson SI, Murray J, McWilliams RR (2007). Celiac disease and chemotherapy. Journal of Clinical Oncology.

[14] Stewart AJ, Southcott BM (2002). Coeliac disease following high-dose chemotherapy. Clin Oncol (R CollRadiol).

[15] Schoenfeld SR, Aronow ME, Leaf RK, Dougan M, Reynolds KL (2020). Diagnosis and Management of Rare Immune-Related Adverse Events. Oncologist.

[16] US Department of Health and Human Services. National Cancer Institute. Common terminology criteria for adverse events (CTCAE).

[17] Rubio-Tapia A, Ludvigsson JF, Brantner TL (2012). The prevalence of celiac disease in the United States. Am J Gastroenterol.

[18] Singh P, Arora A, Strand TA (2018). Global Prevalence of Celiac Disease: Systematic Review and Meta-analysis. Clin Gastroenterol Hepatol.

[19] Freeman HJ (1995). Clinical spectrum of biopsy-defined celiac disease in the elderly. Canadian Journal of Gastroenterology.

[20] Cappello M, Morreale GC, Licata A (2016). Elderly Onset Celiac Disease: A Narrative Review. Clin Med Insights Gastroenteroly.

[21] West J, Logan RF, Smith CJ, Hubbard RB, Card TR (2004). Malignancy and mortality in people with coeliac disease: population based cohort study. BMJ.

[22] Collin P, Vilppula A, Luostarinen L (2018). Review article: coeliac disease in later life must not be missed. Aliment Pharmacol Ther.

[23] Han Y, Chen W, Li P, Ye J (2015). Association Between Coeliac Disease and Risk of Any Malignancy and Gastrointestinal Malignancy: A Meta-Analysis. Medicine (Baltimore).

[24] Lebwohl B, Green PHR, Emilsson L (2021). Cancer Risk in 47,241 Individuals With Celiac Disease: A Nationwide Cohort Study. Clin Gastroenterol Hepatol.

[25] Ilus T, Kaukinen K, Virta LJ, Pukkala E, Collin P (2014). Incidence of malignancies in diagnosed celiac patients: a population-based estimate. Am J Gastroenterol.

[26] Marsh MN (1992). Gluten, major histocompatibility complex, and the small intestine. A molecular and immunobiologic approach to the spectrum of gluten sensitivity ('celiac sprue'). Gastroenterology.

[27] Lee SK, Green PH (2005). Endoscopy in celiac disease. Curr Opin Gastroenterol.

[28] Memeo L, Jhang J, Hibshoosh H (2005). Duodenal intraepithelial lymphocytosis with normal villus architecture: common occurrence in H. pylori gastritis. Mod Pathol.

[29] Patterson ER, Shmidt E, Oxentenko AS (2015). Normal villus architecture with increased intraepithelial lymphocytes: a duodenal manifestation of Crohn disease. Am J Clin Pathol.

[30] Lauwers GY, Fasano A, Brown IS (2015). Duodenal lymphocytosis with no or minimal enteropathy: much ado about nothing?. Mod Pathol.

[31] Corazza GR, Biagi F, Volta U (1997). Autoimmune enteropathy and villus atrophy in adults. Lancet.

[32] Malamut G, Verkarre V, Suarez F (2010). The enteropathy associated with common variable immunodeficiency: the delineated frontiers with celiac disease. Am J Gastroenterol.

[33] Lagana SM, Bhagat G (2019). Biopsy Diagnosis of Celiac Disease: The Pathologist's Perspective in Light of Recent Advances. Gastroenterol Clin North Am.

[34] Maurino E, Capizzano H, Niveloni S (1993). Value of endoscopic markers in celiac disease. Dig Dis Sci.

[35] Dickey W, Hughes D (1999). Prevalence of celiac disease and its endoscopic markers among patients having routine upper gastrointestinal endoscopy. Am J Gastroenterol.

[36] Oxentenko AS, Grisolano SW, Murray JA (2002). The insensitivity of endoscopic markers in celiac disease. Am J Gastroenterol.

[37] Dickey W (2006). Endoscopic markers for celiac disease. Nat Clin Pract Gastroenterol Hepatol.

[38] Abdel-Wahab N, Shah M, Suarez-Almazor ME (2016). Adverse Events Associated with Immune Checkpoint Blockade in Patients with Cancer: A Systematic Review of Case Reports. PLoS One.

[39] Badran YR, Shih A, Leet D (2020). Immune checkpoint inhibitor-associated celiac disease. J Immunother Cancer.

[40] Alsaadi D, Shah NJ, Charabaty A (2019). A case of checkpoint inhibitor-induced celiac disease. J Immunother Cancer.

[41] Walton H, Hopkins S, Shand A (2021). Immunotherapy-induced coeliac disease in curative lung cancer. BMJ Case Rep.

[42] Theodoraki E, Giannarakis M, Tzardi M (2021). Pembrolizumab-induced antiTTG IgA-negative duodenitis treated with gluten withdrawal. Eur J Gastroenterol Hepatol.

[43] Nitenberg G, Raynard B (2000). Nutritional support of the cancer patient: issues and dilemmas. Crit Rev OncolHematol.

[44] Andreyev HJ, Norman AR, Oates J (1998). Why do patients with weight loss have a worse outcome when undergoing chemotherapy for gastrointestinal malignancies?. Eur J Cancer.

[45] Schuppan D, Mäki M, Lundin KEA, Isola J (2021). A Randomized Trial of a Transglutaminase 2 Inhibitor for Celiac Disease. N Engl J Med.

